# Postnatal, ontogenic liver growth accomplished by biliary/oval cell proliferation and differentiation

**DOI:** 10.1371/journal.pone.0233736

**Published:** 2020-05-29

**Authors:** Armanda Szücs, Sándor Paku, Endre Sebestyén, Péter Nagy, Katalin Dezső

**Affiliations:** First Department of Pathology and Experimental Cancer Research, Semmelweis University, Budapest, Hungary; University of Alberta, CANADA

## Abstract

**Introduction:**

The liver is well known for its enormous regenerative capacity. If the hepatocytes are compromised the reserve stem cells can regrow the lost tissue by means of oval cells differentiating into hepatocytes. We were curious whether this standby system was able to compensate for ontogenic liver growth arrested by 2-acetylaminofluorene (AAF) treatment or if it can be influenced by cholic acid, known to promote liver growth in several reactions.

**Methods:**

(i) Four weeks-old (60-70g) male F344 rats were kept on standard chow and treated with solvent only, (ii) others were kept on 0,2% cholic acid (CA) enriched diet, (iii) treated with AAF, or (iiii) given a combination of CA diet and AAF treatment (AAF/CA).

The proliferative response of epithelial cells was characterized by pulse bromodeoxyuridine labelling. The relative gene expression levels of senescence-related factors and bile acid receptors were determined by quantitative real-time polymerase chain reaction analysis.

**Results:**

AAF administration efficiently inhibited the physiological proliferation of hepatocytes in young, male F344 rats after weaning. The activation of stem cells was indicated by the increased proliferation of periportal biliary/oval cells (B/OC). If the rats were fed additionally by cholic acid enriched diet, typical oval cell reaction emerged, subsequently the oval cells differentiated into hepatocytes restituting liver growth. This reaction was mediated by increased production of HGF, IL-6 and SCF by the damaged liver. Moreover, upregulation of FXR expression on B/OC made them competent for bile acids. Our results indicate that endogenous, autocrine mechanisms involved in liver ontogeny are also able to activate the backup regenerative machinery of stem cells.

## Introduction

There are several non-tumorous growth reactions of the hepatic tissue with different origin, regulation and function. We have observed certain differences between structural aspects of postnatal ontogenic liver growth and compensatory hyperplasia induced by surgical partial hepatectomy [1]. While the liver expands exclusively by the enlargement of pre-existent lobules after partial hepatectomy, the generation of new lobules also contributes to physiological growth. The regulation of these reactions is not completely understood, similar and divergent mechanisms are also known [2] e.g. FGF and TGF β signalling seems to be the most important during ontogenesis, while HGF and EGFR ligands are the most important for regenerative growth. There is a well-known back up mechanism of compensatory hyperplasia. If the proliferative capacity of the hepatocytes is compromised the so called oval cells emerge in the periportal areas which regenerate the lost parenchyma by proliferation/differentiation [3]. This tissue reaction is extensively characterized in rodents and shows similarities with certain forms of ductular reaction in human liver [4, 5]. One of the most widely used experimental models of oval cell proliferation/differentiation in rats consists of 2/3 partial hepatectomy (Ph) with continuous AAF administration [6]. The AAF treatment results in a mild periportal biliary cell proliferation, following the surgery these cholangiocytes invade the hepatic parenchyma, change several phenotypic features, and turn into oval cells which later differentiate into hepatocytes [7, 8]. The oval cells are regarded as the progenies of the hepatic stem cells [9] in the context of the cellular hierarchy of the liver.

As far as we know it has not been studied if this alternative mechanism can be elicited by the hindrance of postnatal ontogenesis of the liver. In order to investigate this possibility young rats following weaning were treated by AAF. AAF administration itself caused only moderate periportal spreading of B/OC ductules, but if AAF treatment was complemented with a diet enriched in cholic acid typical, intense B/OC proliferation was induced. The regulation of this growth reaction has been analysed.

## Methods

### Animal experiments

F344 rats were in-house bred. The breeding animals were purchased from Charles-River Laboratories (Écully, France).

Plastic cages (556x334 mm, Animalab, Poznań, Poland) with wood chip bedding, cardboard tubes and paper wool nest material were used for housing. Rats were group-housed (5 rats/per cage) and kept on a 12hr light dark cycle (lights on at 7:00 hours) in a constant temperature (23°C) and humidity (22%). They were provided standard chow (V1535000, SSNIFF, Soest, Germany; 15mm pellets) and water ad libitum.

The experiments were conducted on 4 weeks-old (60-70g) male F344 rats randomly divided into 4 groups: (i) Control animals (n = 15) were kept on standard chow and treated with solvent only, (ii) others (n = 15) were kept on 0,2% CA diet (C1129 Sigma Aldrich, St. Louis, MO, was added to the standard chow by Altromin, Lage, Germany), (iii) treated with AAF (n = 15) (A7015, Sigma-Aldrich, dissolved in 1% methylcellulose, administered daily by gavage, 5mg/kg), or (iiii) given a combination of CA diet and AAF treatment (AAF/CA) (n = 24). Animals were sacrificed on the 3rd, the 7th and the 10th days after the initiation of treatment (n = 5–8 per time points). Each animal was given 200 mg/kg bromodeoxyuridine (BrdU, B5002, Sigma-Aldrich) intraperitoneally 1h before termination. During the experiments the wellbeing (the positive mental state, the ability to achieve successful biological function, the innate behaviours, coping with potentially adverse conditions) and the health (body condition, weight, changes in body shape, posture, fur, facial expression, eyes, ears, nose, mouth, tail) was assessed by the investigators every day.

After humanely sacrificing the animals using cervical dislocation, samples from the livers were taken and fixed in Bouin’s solution for histological examination and the rest were snap-frozen in liquid nitrogen.

The animal study protocols were conducted according to the National Institute of Health (NIH) guidelines for animal care and were approved by the Institutional Animal Care and Use Committee of Semmelweis University (KA-1771).

### Histological analysis

#### Immunohistochemistry

AFP (1:50, A0008, Dako, Glostrup, Denmark) immunostaining was performed on Bouin’s-fixed paraffin embedded samples using the Novolink Polymer Detection System (RE7140-K, Leica Biosystems, Wetzlar, Germany) and DAB (SK-4105, Vector Laboratories, Burlingame, CA) as chromogen. Frozen sections were fixed in methanol for 10 min and incubated at room temperature for 1h with the primary antibodies (OV-6: 1:50, MAB2020, R&D, Minneapolis, MN; SMA: 1:100, M0851, Dako; Desmin: 1:200, PA1-37556, Thermo Fisher Scientific; Laminin:1:200, Z0097, Dako; DLK1: 1:100, AF1144, R&D), then with appropriate secondary antibodies (Alexa Fluor dyes conjugated antibodies, Thermo Fisher Scientific, Waltham, MA).

#### Morphometric analysis

The area occupied by B/OC was measured on OV-6 immunostained frozen sections. Sections were scanned using Pannoramic Confocal system (3D-Histech Ltd., Budapest, Hungary), and evaluated by the ImageJ 1.49k program (NIH, Bethesda, MD).

#### Determination of the BrdU-index

After pre-treating the frozen sections with 2N HCl (10min, at room temperature) the incorporated BrdU was immunostained (BrdU antibody:1:20, 347580, BD Biosciences, Franklin Lakes, NJ). 10.000 hepatocytes and 500 B/OC were counted; the percentage of BrdU-positive cells was given as a result. Nuclei were stained with DAPI (D9542, Sigma-Aldrich).

### Quantitative real-time polymerase chain reaction

#### Microdissected samples

Frozen sections made from the livers of each experimental group (on the 10^th^ day of treatment) were fixed in methanol, stained with RNase-free haematoxylin and dried at room temperature. Laser microdissection of B/OC and hepatocytes (~100.000 μm^2^) was performed by using MMI CellCut laser microdissection (Eching, Germany). Total RNA was isolated by the RNA Aqueous Micro Kit (AM 1931; Life Technologies, Carlsbad, CA). The total amount of isolated RNA was used for reverse transcription.

#### Whole liver samples

Frozen sections from the livers (on the 10^th^ day of the experiment) were collected in lysis buffer. Total RNA was isolated with TRIzol (cat. no. 15596–018; Life Technologies). RNA concentration was measured by NanoDrop 1000 (Thermo Fisher Scientific); 1μg RNA per sample was converted into cDNA.

A high-capacity cDNA reverse transcription kit (4368814; Life Technologies) was used for cDNA synthesis as recommended by the supplier. PCR was performed by the QuantStudio™ 3 System (Thermo Fischer Scientific) sequence detection system, using Life Technologies TaqMan gene expression assays (TGR5: Rn01400316_s1; FXR: Rn00572658_m1; IL-6: Rn01410330_m1; HGF: Rn00566673_m1; SCF: Rn01502851_m1; CTGF: Rn01537279_g1; BIRC5: Rn00574012_m1; IFNG: Rn00594078_m1; TNFR1: Rn00565310_m1) according to the manufacturer’s instructions. Glyceraldehyde-3-phosphate dehydrogenase (GAPDH) was used as endogenous control. All samples were run in triplicate, in a 20 μl reaction volume. Results were obtained as threshold cycle (CT) values. Expression levels were calculated using the ΔC_T_ method. The values were calculated as the mean values of three independent measurements, and the expression levels of mRNA in all samples were defined as a ratio to GAPDH expression.

### Statistical analysis

Data are presented as means ± standard error (SEM) or standard deviation (SD). We used the R statistical environment (version 3.6.0) for all significance test. The statistical significance of difference between groups was analyzed by analysis of variance (ANOVA, one-way) or Welch's two sample t-test (two-sided). Since an elevation in the senescence-associated secretory phenotype (SASP)-factors expression could be hypothesized, so for the analysis of these factors we used the one-sided Welch’s-test with an alternative hypothesis of less expression in the control condition. Results were considered significant at P≤0.05.

## Results

In the present study, we were going to investigate if B/OC can contribute to the ontogenic liver growth in rats. Since the B/OC proliferation and differentiation was defined by the histological changes, the morphology of the livers was thoroughly analysed, followed by immunohistochemical, morphometric, proliferation and gene expression studies.

### The AAF/CA protocol induces typical oval cell proliferation

**Fig 1:**
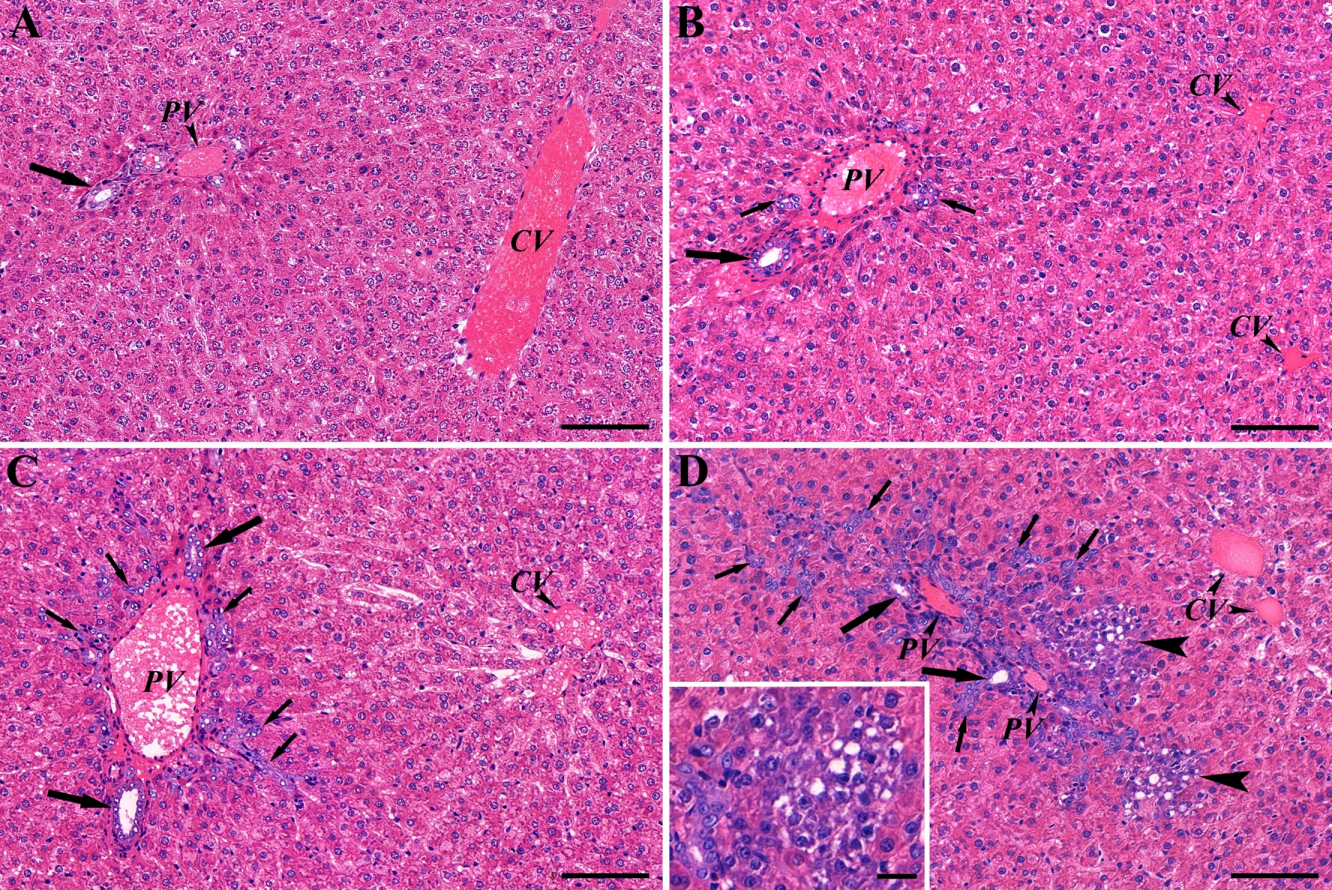
Histological characteristics of the livers after AAF/CA treatment. HE stained sections of control liver (A) and on the 3^rd^ (B), the 7^th^ (C) and the 10^th^ days (D) of the AAF/CA treatment. The combined treatment elicited the gradual expansion of B/OC (arrows) into the parenchyma between 3–10 days and appearance of basophilic small hepatocyte groups at 10 days (large arrowheads on (D)) which were localized closely to B/OC. Inset shows at higher power the small hepatocytes. Some of them contain lipid droplets. Large arrows point at bile ducts. Scale bars: 100 μm, Scale bar for inset 20 μm.

The histological structure of the liver was completely normal in the control (Fig 1A) and CA groups at each time point (S1 Fig). No obvious change was seen in the liver of the AAF treated animals on the 3^rd^ day, but on the 7^th^ and especially on the 10^th^ days the proliferation of B/OC could be seen. Some of the ducts became tortuous, mitotic figures could rarely be seen. However, these altered B/OC ducts were confined to the periportal space, did not enter the parenchyma (S1 Fig).

The number of B/OC remarkably increased on the 3^rd^ day in the AAF/CA group (Fig 1B). Later typical “oval cells” penetrated the limiting plate and infiltrated the hepatic parenchyma (Fig 1C). The B/OC formed ductules spread further on the 10^th^ day. In addition, rows or occasionally small foci of hepatocyte-looking cells emerged with vacuolated, basophilic cytoplasm. These newly formed groups of cells were always spatially associated with the oval cells (Fig 1D).

**Fig 2:**
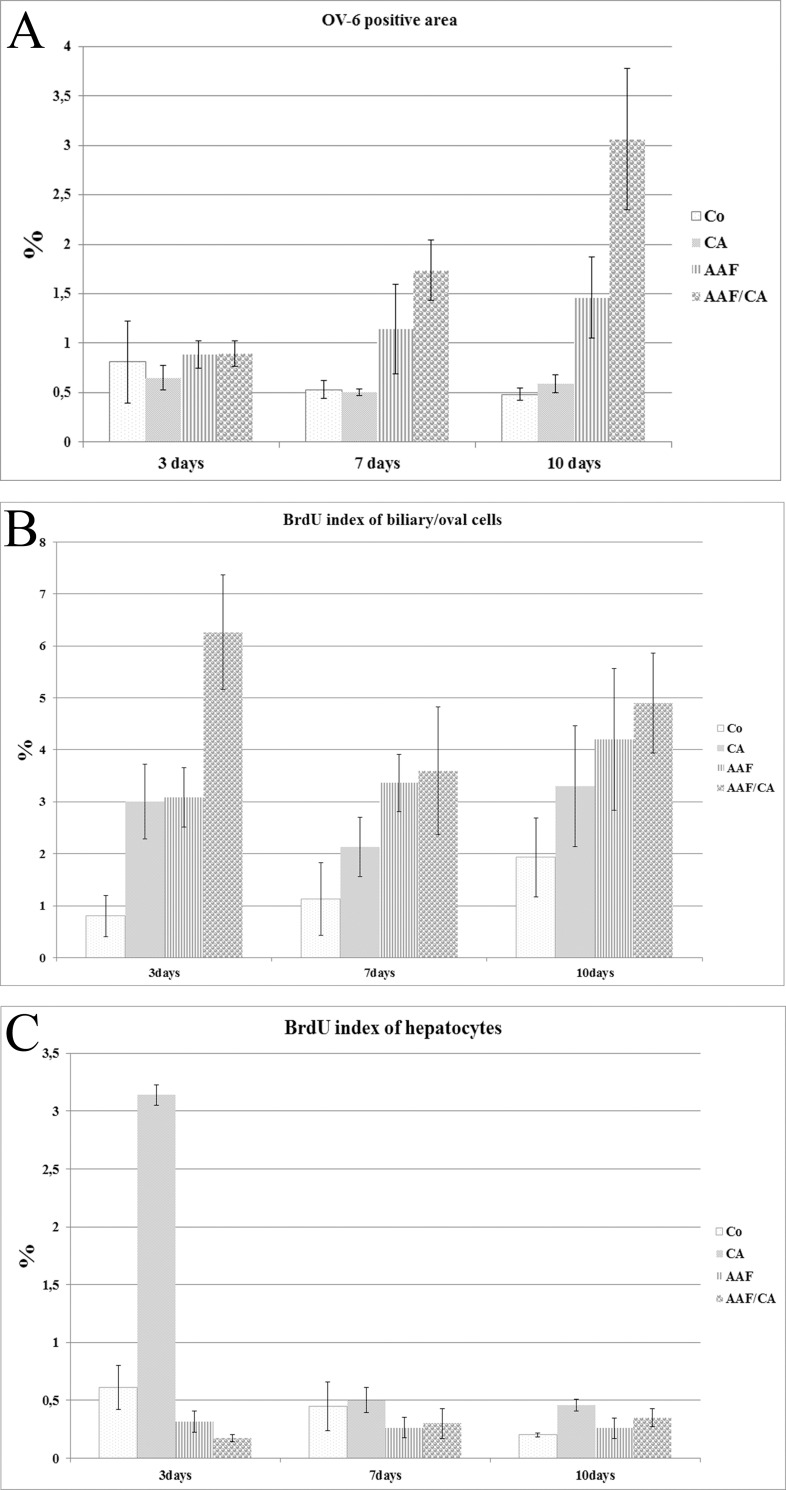
Quantitative analysis of the morphological alterations. A. The extent of OV-6 positive area in the experimental groups. B. The proliferative activity (BrdU-index) of the B/OC. C The proliferative activity (BrdU-index) of the hepatocytes. Data are represented as means ± standard deviation of the mean.

The OV-6 antibody recognizes Keratin 14 and 19 [10]. Although it labels normal cholangiocytes too it is regarded as an oval cell specific marker. The extent of B/OC proliferation can be characterized by the relative area stained by OV6 (S2A and S2B Fig). This parameter gradually, but not significantly declined in the Control and CA fed rats indicating that the area occupied by B/OC decreases with age. However, the OV-6 stained area increased in the two other groups especially in the AAF/CA treated animals (Fig 2A, S1 Table and S2B Fig). Since on the 10^th^ day the emerging, most likely B/OC derived new basophilic hepatocytes (see later) in the AAF/CA rats were OV6 negative the quantitation of OV-6 staining even underestimated the participation of the B/OC at this time-point.

### Oval cell proliferation compensates for the growth inhibition of hepatocytes

Cell proliferation was characterized by the evaluation of BrdU incorporation following pulse labelling (S2C and S2D Fig). Since the cholangiocytes and oval cells could not be easily distinguished, they were evaluated together as one cell-compartment (B/OC) and the percentage of labelled hepatocytes was counted separately.

The ratio of BrdU marking the B/OC was gradually elevating (Fig 2B, S2 Table) while the proliferative activity of hepatocytes declined with time (Fig 2C, S3 Table) in the control group, but these variations did not seem to be biologically important. AAF treatment decreased the BrdU incorporation in the hepatocytes, although the inhibition was far from complete (Fig 2C, S3 Table). Increased BrdU labelling of B/OC serves as explanation for the expansion of this compartment upon AAF administration.

Cholic acid enriched diet resulted in a substantial but temporary increase in proliferative activity of hepatocytes without influencing the behaviour of B/OC. This hump of hepatocyte proliferation was completely eliminated in the AAF/CA group, but the BrdU incorporation of B/OC was significantly higher in the combined group (Fig 2B, S2 Table) at each time point.

### The AAF/CA treatment results in B/OC differentiation

**Fig 3:**
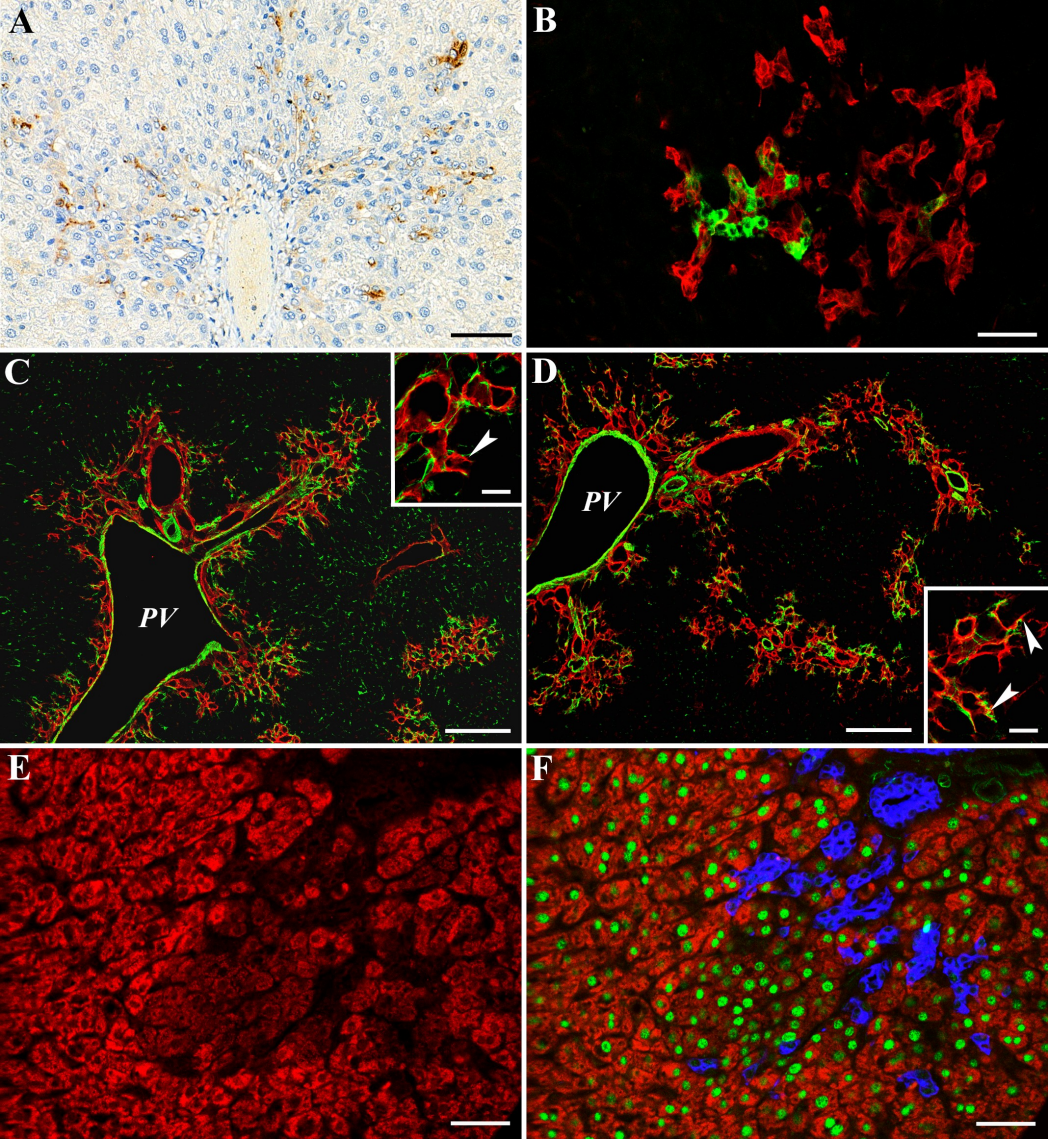
Characterization of bile ducts (oval cells) and small hepatocytes on 10^th^ days of the AAF/CA treatment. A. Bouin’s fixed section stained for AFP. AFP positive B/OC ductules spread from the portal area into the parenchyma. Scale bar: 50 μm. B. Frozen section stained for OV6 (red) and DLK1 (green). Numerous DLK1 positive cells are situated within the OV6 positive B/OC ductules. Scale bar: 50 μm. C. Frozen section stained for laminin (red) and Desmin (green). Laminin positive basement membrane framed ductules accompanied by desmin positive cells spread from the portal area into the parenchyma. Inset shows typical “U” shaped (arrowhead) termination of B/OC ductules on hepatocytes. Desmin positive myofibroblasts are closely associated with the laminin positive basement membrane. PV; Portal vein. Scale bar: 200 μm, Scale bar for inset: 25 μm. D. Frozen section stained for laminin (red) and SMA (green). Laminin positive basement membrane framed ductules accompanied by SMA positive cells spread from the portal area into the parenchyma. Inset as before shows typical “U” shaped (arrowheads) termination of B/OC ductules on hepatocytes. SMA, another established marker of myofibroblasts is also closely associated with the laminin positive basement membrane. PV; Portal vein. Scale bar: 200 μm, Scale bar for inset: 25 μm. E. Frozen section stained with streptavidin-TRITC to detect endogenous biotin. Group of small hepatocytes characterized by low endogenous biotin content are surrounded by native hepatocytes with higher biotin content. Scale bar: 100 μm. F. The same area is visible on E., stained additionally for HNF-4 (green) and OV6 (blue). The nuclei of small hepatocytes with low biotin content are positive for HNF-4. Differentiating small hepatocytes are in close vicinity of the OV6 positive B/OC ductules. Scale bar: 100 μm.

The oval cells show similarities to cholangiocytes, they are most likely descendants of stem cells of the biliary cell compartment. The two cell types can be distinguished by their phenotype and arrangement. All the oval cell characteristic features were present on the cells evolving in the parenchyma of AAF/CA treated rats. They formed ductules with scanty lumen, and they expressed AFP (Fig 3A) and DLK1 (Fig 3B). The ductules were accompanied by desmin/smooth muscle actin positive myofibroblasts (Fig 3C and 3D). The ductules terminated on hepatocytes and were surrounded by laminin containing basement membrane (Fig 3C and 3D). The cut surface of the basement membrane occasionally resulted in a characteristic “U” shape. We observed this special arrangement during liver regeneration in the AAF/Ph protocol [7, 8].

The basophilic cells, which were closely associated with B/OC in the latest time point of AAF/CA treated rats were not anchored on basement membrane. The forthcoming HNF4 staining with the formerly described alterations clearly indicated hepatocytic differentiation. The low endogenous biotin content of the basophilic cells demonstrated by decreased avidin binding distinguished them from pre-existent hepatocytes (Fig 3E and 3F).

### AAF makes stem cells competent for cholic acid

**Fig 4:**
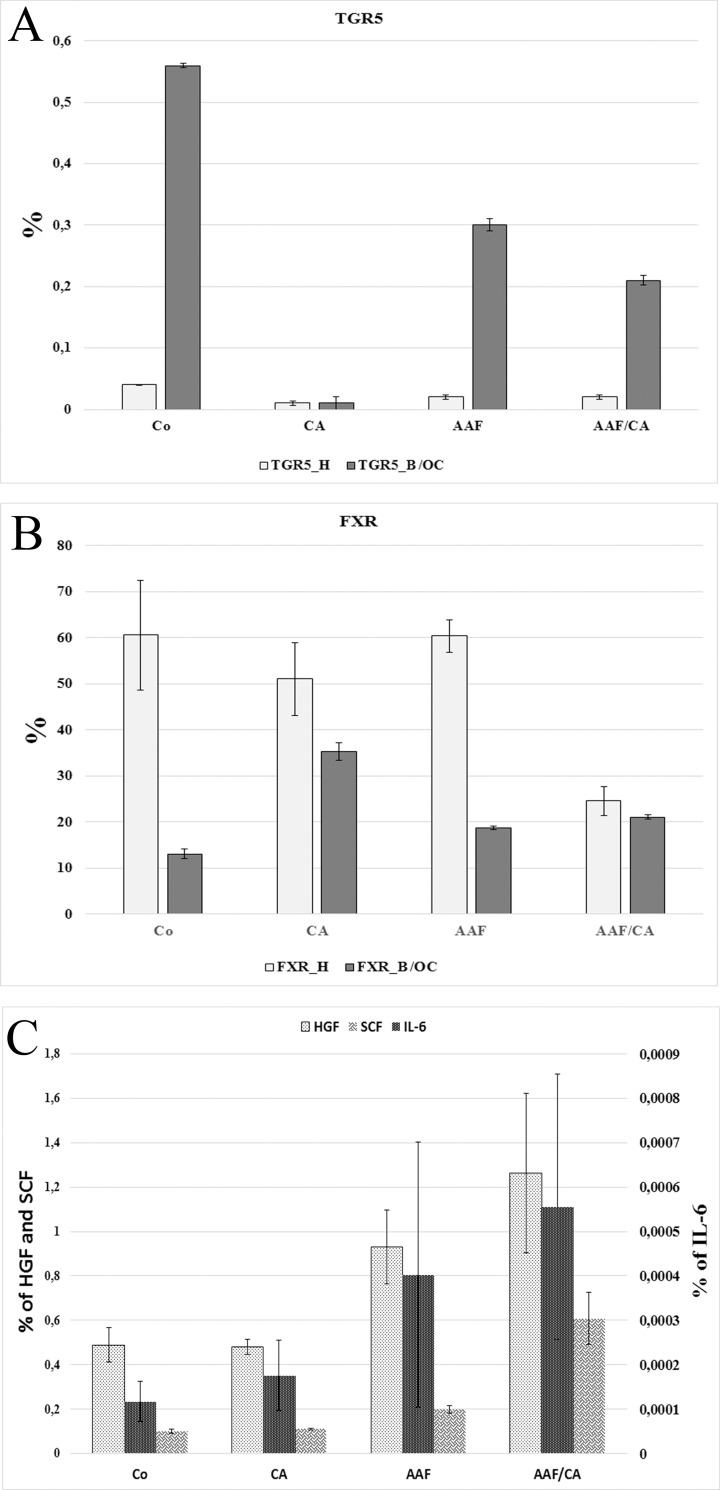
Relative mRNA level of bile acid receptors and senescence associated proteins. A.B. Real-time QRT-PCR analysis of TGR5 (A) and FXR (B) mRNA expression in microdissected hepatocytes (H) and biliary/oval cells (B/OC) on the10^th^ day of the experiment. Bars represent SE. C. Real-time QRT-PCR analysis of IL-6, SCF and HGF mRNA expression in whole liver samples on the 10^th^ day of the experiment. Bars represent SE.

RNA was isolated from microdissected B/OC and hepatocytes separately. The RNAs were probed for the two major bile acid receptors. TGR expression was down regulated in both cell populations in each experimental groups compared to the controls (Fig 4A, S4 Table). However, FXR, which is thought to transduce the proliferative signal of bile acids [11, 12], was upregulated on the B/OC of the AAF/CA rats while its expression decreased on the hepatocytes (Fig 4B, S4 Table).

RNA was isolated from whole liver tissue of each experimental group as well. Real time RT PCR demonstrated significantly increased HGF and SCF expression following AAF treatment (Fig 4C, S5 Table). No such elevation could be detected in the expression of TNFR1, IFN gamma, CTGF and survivin (S6 Table).

## Discussion

AAF was able to efficiently temper the proliferation of hepatocytes in young rapidly growing rats. If such animals were fed cholic acid enriched diet, this growth defect could be compensated by the appearance of small biliary looking cells emerging in the periportal zone and later spreading into the parenchyma. These cells formed ductules surrounded by basement membrane, they expressed AFP, DLK1, they were closely associated with SMA/desmin positive myofibroblasts and eventually differentiated into HNF4 expressing hepatocytes. These are typical features of the hepatic histological reaction, traditionally referred to as oval cell proliferation [13, 7, 14].

Although no lineage tracing was done in our present experiment, the architectural similarities to the AAF/Ph experiment [15, 7] suggested that the oval cells of these young animals also derived from cholangiocytes and differentiated into hepatocytes. The low biotin content of the small hepatocytes also argued against their hepatocytic origin [8]. The function of the oval cells is to rescue the liver parenchyma in the AAF/Ph experiment since the hepatocytes cannot divide and regrow the missing liver mass [15, 8]. It is still not clear what kind of functional failure initiates the regeneration following partial hepatectomy and what mobilizes the stem cells if the hepatocytes fail. Our results suggest that this mechanism is also activated if the physiological liver growth lags behind. As far as we know, this has not been demonstrated before, although ductular reaction was already induced in young animals by severe parenchymal damage brought about by choline deficient diet [16].

Beside the evident agreements, there are subtle differences between these two models of oval cell proliferation. The combination of proliferative drive (Ph) and impaired hepatocyte proliferation (AAF) are sufficient to result intense oval cell proliferation/differentiation in the AAF/Ph model. In our present experiment another factor, cholic acid enriched diet, was necessary to induce oval cell proliferation albeit a less intense one than in the AAF/Ph experiment. The most likely explanation is that there is no such intense activation drive on the stem cells in these young animals. While the liver weight increased 15–20% in the control animals during our 7 days observation period (from 3–10 days) in young rats, the liver weight increased almost 200% in 7 days following 2/3 surgical Ph in adults ones. AAF almost completely blocks hepatocyte proliferation in adult rats, while this blockage is far from complete in young rats, probably due to lower CYP activity that is required to metabolize AAF [17]. After all it is not so surprising that the intensity of oval cell proliferation and the distribution of the differentiating cells are more similar to the response we have seen after low dose AAF treatment in adult rats [18].

Liver regeneration is thought to be mediated by three clusters of networks: cytokines, growth factors and metabolic signals [19]. The most important and intensively studied metabolic compounds, participating in the growth regulation of liver are bile acids. Bile acid signalling is required for normal liver regeneration, its failure decreases liver regeneration after Ph [11, 12]. The growth promoting effect of cholic acid has become obvious in the present experimental system as well. Cholic acid enriched diet itself [20] resulted in an intense increase in hepatocytic BrdU incorporation. This response was obvious in young rats in our CA group. However, this reaction was almost completely blocked by the additional AAF treatment. What is more, the cell proliferation, serving the physiological growth of the liver shifted to oval cells. There are two major bile acid receptors in the liver farnesoid X receptor (FXR) and G-protein-coupled bile acid receptor (TGR5). Bile acids influence liver regeneration through FXR [11, 12] while TGR5 signals distinct functions [12]. The switch we have experienced in FXR expression, down regulation on hepatocytes and up regulation on B/OC indicates that this altered FXR expression might have contributed to the induction of stem cell driven regeneration.

RT-PCR from whole liver RNA revealed increased HGF, IL-6 and SCF expression in the AAF treated rats, while no such elevation could be detected in the expression of TNFR1 and IFNg. HGF is probably the most potent growth factor driving [9, 21] the proliferation of hepatocytes and oval cells and such function is also known for IL-6 [22] and SCF [23]. These cytokines are also synthetized by senescent cells as components of the senescent associated secretory phenotype [24, 25]. AAF triggers stress induced senescence in hepatocytes [26]. There are other experimental models when stem cell derived ductular or oval cell reaction is induced by the senescence of the hepatocytes [27, 28]. The combination of senescence related growth factor production and the upregulation of FXR on B/OC might be major drivers of the compensatory expansion of B/OC in our AAF/CA experimental model.

Hippo signalling and YAP activation are also known to be important determinants of liver growth and size [29, 30] and bile acids can activate YAP [31], but we could not detect increased steady state level of established YAP target genes (CTGF, survivin). We failed to detect increased production of IFNg, TNFR1, which are considered senescence-related factors [24]. However, these results support, that changes of HGF, Il-6, and SCF expression levels are indeed related to senescence.

In conclusion, we describe a new mechanism, the inverse expression of FXR on hepatocytes and B/OC, which can contribute to the activation of hepatic stem cells when the hepatocytes are compromised. This mechanism is activated when the hepatocyte proliferation is hindered during the postnatal physiological growth resulting in a stem cell proliferation/differentiation mediated liver growth. The arousal of the hepatic stem cell compartment may inflict adverse consequences e.g. fibrosis, tumorigenesis. Therefore, the long-term follow-up of these animals, whose livers are partly grown by the participation of the stem cell compartment would be very important.

## Supporting information

S1 Fig Histological characteristics of the livers after continuous cholic acid or AAF treatment. (TIF)

S2 Fig Extent of B/OC compartment and BrdU incorporation in the AAF/CA groups. (TIF)

S1 File Legend to the S1 and S2 Figs. (DOCX)

S1 Table Results of the analysis of variance (one-way ANOVA) regarding the area percentage of OV-6 staining. (DOCX)

S2 Table Results of the analysis of variance (one-way ANOVA) regarding the BrdU-index of the biliary/oval cells. (DOCX)

S3 Table Results of the analysis of variance (one-way ANOVA) regarding the BrdU-index of the hepatocytes. (DOCX)

S4 Table Results of Welch’s Two Sample t-test (two sided) used for the statistical analysis of QRT-PCR analysis obtained from LCM samples. (DOCX)

S5 Table Results of Welch’s Two Sample t-test (one-sided, with an alternative hypothesis of less expression in Control condition) used for the statistical analysis of QRT-PCR analysis obtained from whole liver samples. (DOCX)

S6 Table Relative mRNA level of YAP target genes (survivin and Connective Tissue Growth Factor (CTGF)) and senescence-associated factors (IFNg, TNFR1). (DOCX)
