# Supplementary figures and images for: Postnatal, ontogenic liver growth accomplished by biliary/oval cell proliferation and differentiation
Source: PLoS One. 2020 May 29;15(5):e0233736. doi: 10.1371/journal.pone.0233736 (PMC7259787; doi:10.1371/journal.pone.0233736)

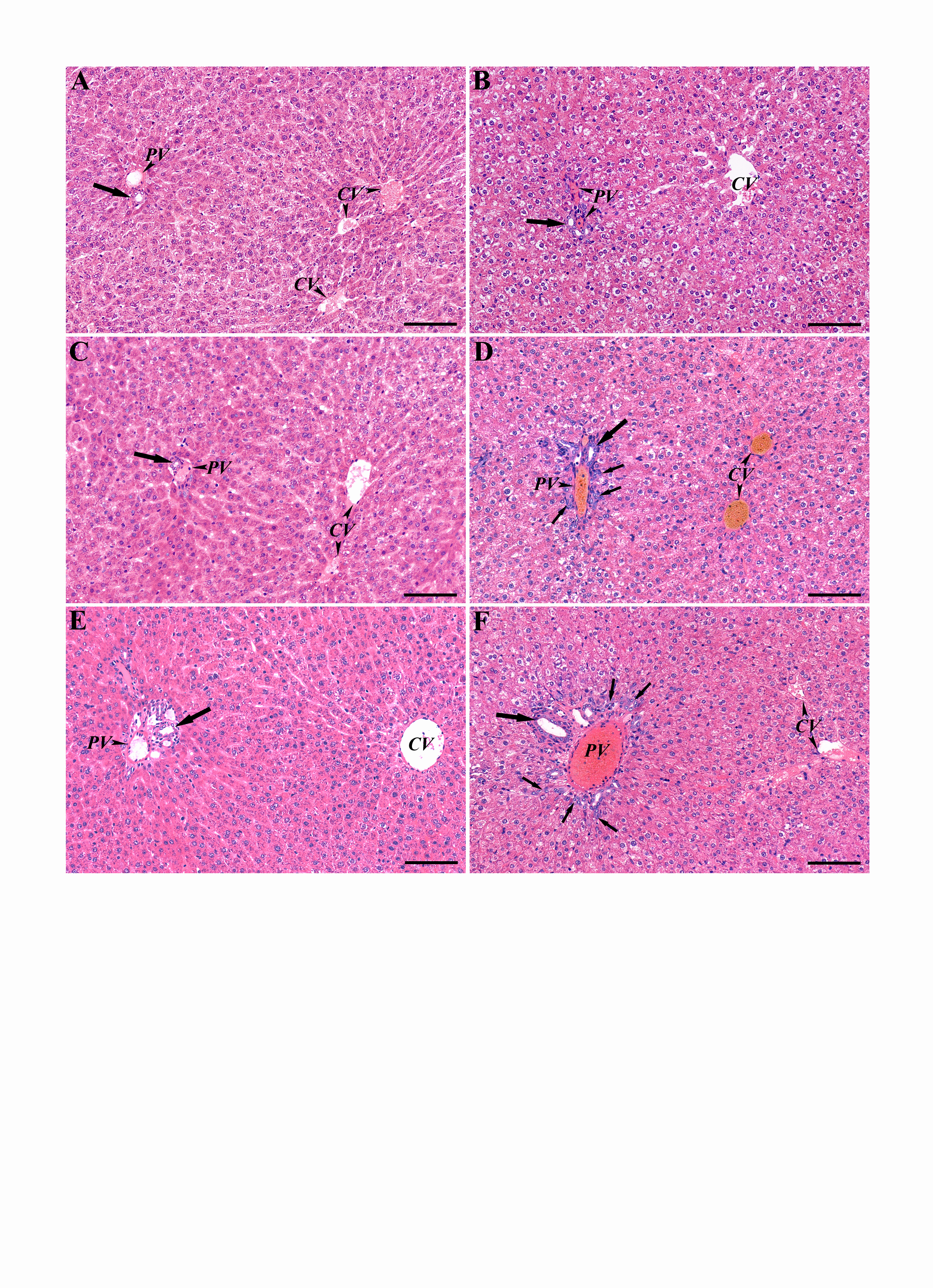

Supplement: S1 Fig — (TIF) [file pone.0233736.s001.tif]

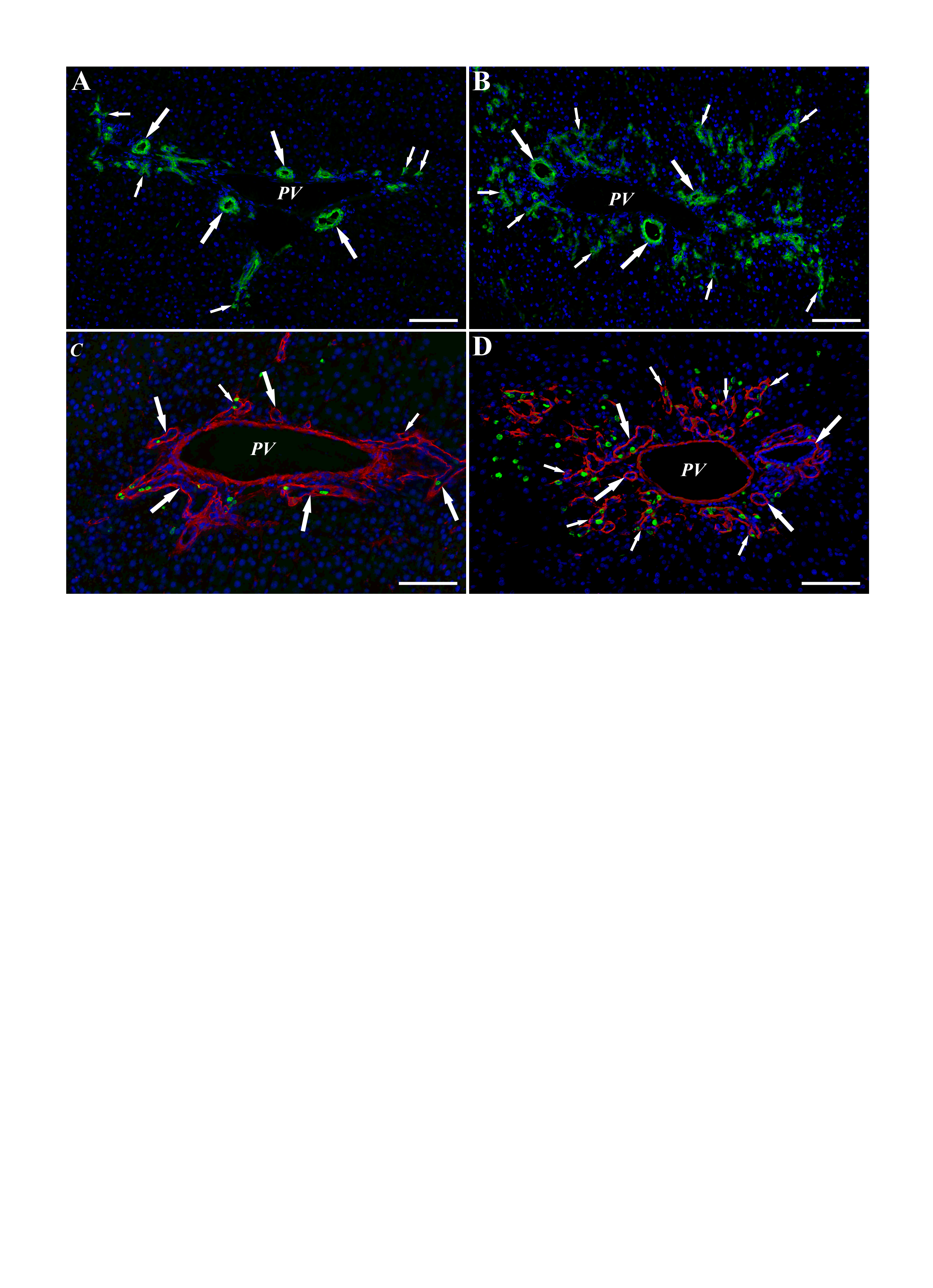

Supplement: S2 Fig — (TIF) [file pone.0233736.s002.tif]
