# Supplementary material for: Postnatal, ontogenic liver growth accomplished by biliary/oval cell proliferation and differentiation
Source: PLoS One. 2020 May 29;15(5):e0233736. doi: 10.1371/journal.pone.0233736 (PMC7259787; doi:10.1371/journal.pone.0233736)
Supplement: S1 File — (DOCX) [file pone.0233736.s003.docx]

**Supporting Figure 1. Histological characteristics of the livers after continuous cholic acid or AAF treatment.**

HE sections of the livers after continuous cholic acid treatment for 3 days (A), for 7 days (C), for 10 days (E) and AAF treatment for 3 days (B), for 7 days (D), for 10 days (F). Cholic acid treatment caused no architectural changes in the liver. AAF treatment (7, 10 days) caused mild periportal oval cell proliferation (arrows). Large arrows point at bile ducts. PV; Portal vein, CV, Central vein. Scale bars: 100 µm.
