# Supplementary material for: Postnatal, ontogenic liver growth accomplished by biliary/oval cell proliferation and differentiation
Source: PLoS One. 2020 May 29;15(5):e0233736. doi: 10.1371/journal.pone.0233736 (PMC7259787; doi:10.1371/journal.pone.0233736)
Supplement: S1 Table — (DOCX) [file pone.0233736.s004.docx]

**Supporting Table 1. Results of the analysis of variance (one-way ANOVA) on the area percentage of OV-6 staining.**

| *Days* | *Comparison* | *p-value* |
| --- | --- | --- |
| Day3 | CA vs. Control | 0,563 |
|  | AAF vs. Control | 0,727 |
|  | AAF/CA vs. Control | 0,758 |
| Day7 | CA vs. Control | 0,621 |
|  | AAF vs. Control | 0,065 |
|  | **AAF/CA vs. Control** | **0,0001** |
| Day10 | CA vs. Control | 0,122 |
|  | **AAF vs. Control** | **0,007** |
|  | **AAF/CA vs. Control** | **0,0003** |

p-values in bold are considered statistically significant.
