# Supplementary material for: Postnatal, ontogenic liver growth accomplished by biliary/oval cell proliferation and differentiation
Source: PLoS One. 2020 May 29;15(5):e0233736. doi: 10.1371/journal.pone.0233736 (PMC7259787; doi:10.1371/journal.pone.0233736)
Supplement: S2 Table — (DOCX) [file pone.0233736.s005.docx]

**Supporting Table 2. Results of the analysis of variance (one-way ANOVA) on the BrdU-index of the biliary/oval cells.**

| *Days* | *Comparison* | *p-value* |
| --- | --- | --- |
| Day 3 | **CA vs. Control** | **0,009** |
|  | **AAF vs. Control** | **0,001** |
|  | **AAF/CA vs. Control** | **0,001** |
| Day 7 | CA vs. Control | 0,129 |
|  | **AAF vs. Control** | **0,002** |
|  | **AAF/CA vs. Control** | **0,011** |
| Day 10 | CA vs. Control | 0,24 |
|  | **AAF vs. Control** | **0,042** |
|  | **AAF/CA vs. Control** | **0,001** |

p-values in bold are considered statistically significant.
