# Supplementary material for: Postnatal, ontogenic liver growth accomplished by biliary/oval cell proliferation and differentiation
Source: PLoS One. 2020 May 29;15(5):e0233736. doi: 10.1371/journal.pone.0233736 (PMC7259787; doi:10.1371/journal.pone.0233736)
Supplement: S3 Table — (DOCX) [file pone.0233736.s006.docx]

**Supporting Table 3. Results of the analysis of variance (one-way ANOVA) on the BrdU-index of the hepatocytes.**

| *Days* | *Comparison* | *p-value* |
| --- | --- | --- |
| Day 3 | **CA vs. Control** | **3,566E-05** |
|  | **AAF vs. Control** | **0,024** |
|  | **AAF/CA vs. Control** | **0,018** |
| Day 7 | CA vs. Control | 0,771 |
|  | AAF vs. Control | 0,119 |
|  | AAF/CA vs. Control | 0,185 |
| Day 10 | **CA vs. Control** | **8,386E-05** |
|  | AAF vs. Control | 0,332 |
|  | **AAF/CA vs. Control** | **0,019** |

p-values in bold are considered statistically significant.
