# Supplementary material for: Postnatal, ontogenic liver growth accomplished by biliary/oval cell proliferation and differentiation
Source: PLoS One. 2020 May 29;15(5):e0233736. doi: 10.1371/journal.pone.0233736 (PMC7259787; doi:10.1371/journal.pone.0233736)
Supplement: S4 Table — (DOCX) [file pone.0233736.s007.docx]

**Supporting Table 4. Results of Welch’s Two Sample t-test (two sided) used for the statistical analysis of QRT-PCR analysis obtained from LCM samples.**

| *Gene* | *Cell_type* | *Comparison* | *p-value* |
| --- | --- | --- | --- |
| **TGR** | **H** | **Control vs. CA** | **0,011** |
| **TGR** | **H** | **Control vs. AAF** | **0,041** |
| TGR | H | Control vs. AAF/CA | 0,085 |
| **TGR** | **B/OC** | **Control vs. CA** | **0,007** |
| **TGR** | **B/OC** | **Control vs. AAF** | **0,0009** |
| **TGR** | **B/OC** | **Control vs. AAF/CA** | **8,74E-05** |
| FXR | H | Control vs. CA | 0,301 |
| FXR | H | Control vs. AAF | 0,955 |
| **FXR** | **H** | **Control vs. AAF/CA** | **0,028** |
| **FXR** | **B/OC** | **Control vs. CA** | **0,001** |
| **FXR** | **B/OC** | **Control vs. AAF** | **0,04** |
| **FXR** | **B/OC** | **Control vs. AAF/CA** | **0,02** |

p-values in bold are considered statistically significant.
