# Supplementary material for: Postnatal, ontogenic liver growth accomplished by biliary/oval cell proliferation and differentiation
Source: PLoS One. 2020 May 29;15(5):e0233736. doi: 10.1371/journal.pone.0233736 (PMC7259787; doi:10.1371/journal.pone.0233736)
Supplement: S5 Table — (DOCX) [file pone.0233736.s008.docx]

**Supporting Table 5. Results of Welch’s Two Sample t-test (one sided, with an alternative hypothesis of less expression in Control condition) used for the statistical analysis of QRT-PCR analysis obtained from whole liver samples.**

| *Gene* | *Comparison* | *p-value* |
| --- | --- | --- |
| HGF | Control vs. CA | 0,49 |
| **HGF** | **Control vs. AAF** | **0,03** |
| **HGF** | **Control vs. AAF/CA** | **0,03** |
| SCF | Control vs. CA | 0,23 |
| **SCF** | **Control vs. AAF** | **0,003** |
| **SCF** | **Control vs. AAF/CA** | **0,002** |
| IL-6 | Control vs. CA | 0,26 |
| IL-6 | Control vs. AAF | 0,37 |
| **IL-6** | **Control vs. AAF/CA** | **0,01** |

p-values in bold are considered statistically significant.
