# Supplementary material for: Postnatal, ontogenic liver growth accomplished by biliary/oval cell proliferation and differentiation
Source: PLoS One. 2020 May 29;15(5):e0233736. doi: 10.1371/journal.pone.0233736 (PMC7259787; doi:10.1371/journal.pone.0233736)
Supplement: S6 Table — (DOCX) [file pone.0233736.s009.docx]

**Supporting Table 6. Relative mRNA level of YAP target genes (Birc5 (surviving) and connective tissue growth factor (CTGF)) and senescence associated factors (IFNg, TNFR1).**

|  | Birc5 (survivin) | CTGF | IFNg | TNFR1 |
| --- | --- | --- | --- | --- |
| Control | 10,41+/-3,46 | 0,44+/-0,15 | 0,001+/-0,0005 | 0,18+/-0,05 |
| CA | 6,3+/-1,42 | 0,29+/-0,005 | 0,0009+/-5,7E-05 | 0,23+/-0,15 |
| AAF | 8,05+/-5,05*(vs.C) | 0,46+/-0,07 | 0,001+/-0 | 0,14+/-0,07 |
| AAF/CA | 10,6+/-6,69 | 0,57+/-0,14 | 0,0009+/-0,0001 | 0,3+/-0,06*(vs.C) |

Data are represented as means ± standard deviation of the mean. p>0,05 (* p<0,05)
